# Aberrant brain topological organization and granger causality connectivity in Parkinson’s disease with impulse control disorders

**DOI:** 10.3389/fnagi.2024.1364402

**Published:** 2024-04-25

**Authors:** Caiting Gan, Heng Zhang, Huimin Sun, Xingyue Cao, Lina Wang, Kezhong Zhang, Yongsheng Yuan

**Affiliations:** ^1^Department of Neurology, The First Affiliated Hospital of Nanjing Medical University, Nanjing, China; ^2^Jiangsu Key Laboratory of Neurodegeneration, Nanjing Medical University, Nanjing, China

**Keywords:** impulse control disorders, graph theory, white matter, granger causality analyses, Parkinson’s disease

## Abstract

**Introduction:**

Impulse control disorders (ICDs) refer to the common neuropsychiatric complication of Parkinson’s disease (PD). The white matter (WM) topological organization and its impact on brain networks remain to be established.

**Methods:**

A total of 17 PD patients with ICD (PD-ICD), 17 without ICD (PD-NICD), and 18 healthy controls (HCs) were recruited. Graph theoretic analyses and Granger causality analyses were combined to investigate WM topological organization and the directional connection patterns of key regions.

**Results:**

Compared to PD-NICD, ICD patients showed abnormal global properties, including decreased shortest path length (Lp) and increased global efficiency (Eg). Locally, the ICD group manifested abnormal nodal topological parameters predominantly in the left middle cingulate gyrus (MCG) and left superior cerebellum. Decreased directional connectivity from the left MCG to the right medial superior frontal gyrus was observed in the PD-ICD group. ICD severity was significantly correlated with Lp and Eg.

**Discussion:**

Our findings reflected that ICD patients had excessively optimized WM topological organization, abnormally strengthened nodal structure connections within the reward network, and aberrant causal connectivity in specific cortical– limbic circuits. We hypothesized that the aberrant reward and motor inhibition circuit could play a crucial role in the emergence of ICDs.

## 1 Introduction

Impulse control disorders (ICDs) are repetitive reward-seeking behaviors that are increasingly recognized as a serious complication of Parkinson’s disease (PD). Patients exhibited novelty-seeking personalities and excessive desire for risk-taking behaviors (Santangelo et al., 2017). The cardinal presentations of ICD include pathologic gambling (PG), hypersexuality (HS), binge eating (BE), and compulsive shopping (CS). More recently, it has been reported that the 5-year cumulative incidence of ICDs is approximately 50% (Corvol et al., 2018). ICDs in PD are considered to develop in response to dopamine replacement therapy (DRT) and in particular to increased exposure to dopamine agonist, as well as the interaction with underlying PD pathophysiology and possibly personality traits to manifest as particular behavioral phenotypes (Houeto et al., 2016; Antonini et al., 2017; Dawson et al., 2018; Baig et al., 2019). This disturbance has devastating psychosocial and financial consequences for the person concerned and their family. At present, the pathophysiology of ICD has not been elucidated. A couple of probable mechanisms that may be responsible for ICD have been proposed, including reduced inhibition, reward pathways dysfunction, prompt decision-making without ample evidence, strengthened novelty seeking, poor learning ability from negative feedback, and a preference for immediate, smaller rewards over delayed, and larger rewards (Drew et al., 2020). However, there is no universal agreement on which of these mechanisms plays the most critical role. Given that dopamine has disparate influence on brain function, the possibility remains that several different mechanisms may work simultaneously.

Pathological evidence manifested that accumulating alpha-synuclein was implicated in ICDs and could lead to white matter (WM) alterations in the form of Lewy neurite (Braak et al., 2003; Foroud et al., 2007). With DTI technology, we can non-invasively detect the integrity of WM microstructure. The existing DTI studies revealed several abnormalities in the WM structure in ICD patients (van Timmeren et al., 2017; Vermeij et al., 2018; Sparks et al., 2020; Dolatshahi et al., 2021). The graph theoretic analyses described the WM tracts as edges and the brain regions as nodes to investigate the large-scale brain structural networks (Wang et al., 2019). Using the graph method, it is possible to measure the information exchange between distributed brain regions and specialized information communication within densely interconnected groups of regions, namely the integration and separation of WM structural networks and the importance of a brain region. A potential theory was that the disrupted microstructural integrity of WM would influence structural network topological organization (Cai et al., 2022). Therefore, we speculated that the topological properties of ICD patients might be altered. Until now, the research on the WM topological properties of PD-ICD has been limited.

Furthermore, the abnormal WM microstructure of PD-ICD patients may incorporate important clues to comprehend the altered functional connectivity between networks. The information flow was important to have a comprehensive understanding of the role of the key nodes. Granger causality analysis (GCA) implements a statistical, predictive notion of causality, and provides a hypothesis-driven approach to assessing the impact of one region’s activity on another region via top-down mechanisms (Seth et al., 2015). The GCA can be used to clarify the integration of functionally specialized regions and offer insight into the directionality of information transfer.

We hypothesized that the disrupted WM topological organization and abnormal directional connectivity of the significant nodes could be potential mechanisms for PD patients developing ICD. To verify this conjecture, we explored the WM structural topological properties of PD-ICD using the graph theoretical analysis. Moreover, the GCA was utilized to investigate the hierarchical signal processing and causal connectivity patterns between regions with significant graph parameters and other brain areas.

## 2 Materials and methods

### 2.1 Subjects

We recruited 52 participants from the Neurology Department of the First Affiliated Hospital of Nanjing Medical University, Nanjing, China. A total of 17 PD patients with ICD (PD-ICD), 17 PD patients without ICD (PD-NICD), and 18 healthy controls (HCs) were recruited consecutively. Three groups were matched by age, gender, and education level. The inclusion criteria for the study were as follows: (1) confirmed diagnosis of idiopathic PD according to the Movement Disorder Society clinical diagnostic criteria (Postuma et al., 2015); (2) administration of dopaminergic medication for at least 12 months and maintenance of steady doses for at least 2 months preceding the examination; (3) absence of comorbid other neurological disorder, affective disease, psychotic diseases, or cognitive deficits [Mini-Mental State Examination (MMSE) under 27]; and (4) no contraindications for MRI scans. ICD was identified using the Questionnaire for Impulsive-Compulsive Disorders in Parkinson’s Disease (QUIP) (Weintraub et al., 2009). ICD-positive patients were interviewed by experienced neuropsychologists to confirm the clinical phenotype (PG, HS, BE, or CS). Furthermore, ICD severity was assessed using the QUIP Rating Scale (QUIP-RS). Moreover, each ICD individual suffered from at least one ICD subtype with a QUIP-RS score above the threshold (Weintraub et al., 2012).

This research was ratified by the ethics committee of the First Affiliated Hospital of Nanjing Medical University. All participants provided signed consent before participating in the study.

### 2.2 Demographic and clinical evaluation

All patients received detailed assessments in ON-medication conditions. Data pertaining to age, gender, period of education, PD onset age, and the course of illness were collected. The Barratt impulsiveness scale (BIS) was used to evaluate impulsiveness as a personality trait. PD severity and stage were examined using the Unified Parkinson’s Disease Rating Scale-III (UPDRS-III) and Hoehn and Yahr stage (H–Y stage) scale, respectively. Medication is calculated as levodopa equivalent dose (LEDD_TOTAL_) and dopamine agonist equivalent dose (LEDD_DA_). As additional measures of evaluating mental symptoms, the Hamilton Anxiety Scale (HAMA) and the Hamilton Depression Scale-24 (HAMD-24) were applied. The MMSE and Frontal Assessment Battery (FAB) scales were administered to screen global cognition and executive function.

### 2.3 MRI acquisition and preprocessing

MRI scans were acquired from all PD patients while their dopaminergic medications were active and their symptoms were well controlled. Brain MRI scans were collected using a 3.0 T Siemens MAGNETOM Verio whole-body MRI system (Siemens Medical Solutions, Germany) with eight-channel, phase-array head coils. T1-weighted anatomical images covering the whole brain, the DTI data, and the resting-state functional images were obtained. Detailed parameters of the MRI scans are provided in Supplementary Material.

The DTI data preprocessing procedures were summarized as follows: brain extraction, realignment, eddy current, motion artifact correction, fractional anisotropy (FA) calculation, and diffusion tensor tractography. To construct whole-brain fiber tractography, the deterministic trachographic method based on Fiber Assignment by Continuous Tracking (FACT) algorithm was used (Nigro et al., 2016). The primary rs-fMRI data preprocessing steps were as follows: ① removal of the first 10 time points; ② slice timing correction; ③ realignment; ④ spatial normalization using DARTEL; ⑤ spatially smoothing; ⑥ nuisance signal regression; and ⑦ temporal filtering and linearly detrending.

### 2.4 Brain network construction and network parameter calculation

Using the anatomical automatic labeling (AAL) atlas, the whole brain was parcellated into 116 regions of interest (ROIs). Through affine transformation, individual FA maps in native space were co-registered to their corresponding T1-weighted images. Afterward, T1-weighted images were non-linearly normalized to the ICBM152 T1 template. After the above registration, each subject’s inverse transformation parameter was applied to the AAL temple, thus generating the corresponding subject-specific AAL regions. Each AAL region represented a node of a structural brain network. Interconnections between regions were considered the edges of the WM structural network, and the edge was defined only if at least 10 WM fibers were interconnected. Ultimately, a symmetric 116 × 116 structural connectivity matrix for each subject was constructed.

All topological properties were investigated using GRETNA^1^ and visualized through BrainNet Viewer.^2^ The global network architecture was characterized by local efficiency (Eloc), clustering coefficient (Cp), global efficiency (Eg), small-world index (σ), and shortest path length (Lp). Eloc computed specialization, clustering, and the fault-tolerant capacity of the network. Cp reflected the efficiency of information communication between local areas. Eg measured the capacity of transferring parallel information and the efficiency of exchanging information over the whole brain. σ represented the small-world property of the network. By measuring the average nodal shortest Lp, Lp characterized the overall information transmission (Zhou et al., 2021). Four nodal parameters describing local properties were also calculated [nodal local efficiency (NLe), nodal efficiency (Ne), nodal clustering coefficient (NCp), and nodal shortest path length (NLp)] to assess the centrality of a node (Gou et al., 2018).

### 2.5 Voxel-wise granger causality analysis

The voxel-wise GCA was utilized to assess the causal influence between seeds and each voxel within the whole brain, conducted using the rsHRF toolbox^3^ based on statistical parametric mapping (SPM12; Wu et al., 2021).^4^ The times series of the key nodes based on nodal parameters analyses [left middle cingulate gyrus (MCG) and left superior cerebellum (cerebellum 3), see below] were defined as the seed time series. To improve normality, the GCA maps were z-transformed.

### 2.6 Statistical analyses

Demographic and clinical characteristics of all subjects were compared using the chi-square test, two-sample t-test, Mann–Whitney test, one-way analysis of variance (ANOVA), or Kruskal–Wallis test, as appropriate. SPSS 20.0 software was used for data analysis, and statistical significance was set at a *p*-value of <0.05.

To detect the group differences in network parameters, we applied the one-way analyses of covariance (ANCOVA), adjusting age, sex, education level, HAMA scores, HAMD-24 scores, and MMSE scores. For nodal parameters, a *p*-value of <0.05 corrected using Bonferroni was regarded as significant for multiple comparisons. Bonferroni correction was conducted for further between-group differences if significant differences were detected in global or nodal properties. All calculations were performed using the MATLAB toolbox of GRETNA.

For each PD subgroup, the mean z-values of the seed time series to the time course of voxels within the whole brain (the X-to-Y effects) and the time course of voxels within the whole brain to seed time series maps (the Y-to-X effects) were calculated. To discover the differences in the Granger causality maps of key nodes, two-sample t-tests were performed with the same covariates described above. Statistical significance was ascertained using AlphaSim correction^5^ with the cluster-level significance threshold of *p*-value of <0.05 and voxel-level *p*-value of <0.01, determined by a Monte Carlo simulation.

For the topological parameters and causal connectivity with significant inter-group differences, partial correlations were conducted between these properties and the severity of ICD symptoms in PD patients, controlling for age, sex, education level, HAMA scores, HAMD-24 scores, and MMSE scores. A false discovery rate (FDR) corrected *p*-value of <0.05 was used as the threshold for the significance level.

## 3 Results

### 3.1 Demographic and clinical variables

Ultimately, 52 participants (17 with PD-ICD, 17 with PD-NICD, and 18 HCs) were enrolled. Table 1 summarizes the results for the sociodemographic, clinical, and neuropsychological variables among the groups. No significant differences were identified in the field of demographic variables (age, gender, or educational level) and clinical manifestation (disease duration, age at onset, UPDRS-III, H–Y stage, LEDD_TOTAL_, LEDD_DA_, and BIS scores). Regarding mood symptoms, higher scores of HAMA and HAMD-24 were seen in the PD-ICD group relative to HCs (all *p* < 0.001). ICD patients exhibited higher QUIP-RS scores than PD-NICD (p < 0.001). Notably, 16 patients in the PD-ICD group showed a single ICD, and 1 case had multiple ICD symptoms. CS was the most frequent disorder (7/17, 41.2%), followed by BE (4/17, 23.5%), HS (3/17, 17.6%), PG (2/17, 11.8%), and BE+CS (1/17, 5.9%).

**Table 1:** Demographic and clinical characteristics of the studied groups.

Variable	PD-ICD (*n* = 17)	PD-NICD (*n* = 17)	HCs (*n* = 18)	*p*-value	*Post hoc* (Bonferroni correction)
Age (y)	58.9 ± 9.6	60.1 ± 5.9	64.1 ± 5.1	0.055^c^	
Sex (M/F)	11/6	9/8	14/4	0.303^a^	
Education (y)	12.1 ± 2.7	11.1 ± 2.3	11.9 ± 3.8	0.599^c^	
Disease duration (y)	9.5 ± 5.4	6.8 ± 2.4	-	0.075^e^	
Age at onset (y)	52.2 ± 7.8	57.1 ± 7.3	-	0.071^e^	
UPDRS part 3	35.5 ± 11.2	35.4 ± 11.4	-	0.976^e^	
H–Y stage	2.3 ± 0.8	2.2 ± 0.6	-	0.810^d^	
LEDD_TOTAL_ (mg)	765.3 ± 329.2	619.6 ± 196.6	-	0.127^e^	
LEDD_DA_ (mg)	86.8 ± 54.7	76.8 ± 44.7	-	0.567^e^	
QUIP-RS total score	12.9 ± 6.6	0	-	<0.001^d,***^	
BIS	34.4 ± 17.9	31.5 ± 14.5	-	0.601^e^	
MMSE	28.5 ± 1.2	28.1 ± 1.1	29.0 ± 1.2	0.067^c^	
HAMA	14.7 ± 9.2	12.2 ± 5.5	2.6 ± 3.4	<0.001^c,***^	PD-ICD > HCs (*p* < 0.001^***^) PD-NICD > HCs (*p* < 0.001^***^)
HAMD-24	12.4 ± 9.1	10.9 ± 7.0	1.7 ± 2.8	<0.001^c,***^	PD-ICD > HCs (*p* < 0.001^***^) PD-NICD > HCs (*p* < 0.001^***^)
FAB	16.1 ± 2.1	16.0 ± 3.0	-	0.823^c^	

Values are presented as the mean ± standard deviation. PD, Parkinson’s disease; ICDs, impulse control disorders; HCs, healthy controls; M, male; F, female; y, year; UPDRS, Unified Parkinson’s disease rating scale; H–Y stage, Hoehn and Yahr rating scale; LEDD_TOTAL_, total levodopa equivalent daily dose; LEDD_DA_, levodopa equivalent daily dose of dopamine agonist; DA, dopamine agonist; QUIP-RS, Questionnaire for Impulsive Compulsive Disorders in Parkinson’s Disease-Rating Scale; BIS, Barratt Impulsiveness Scale; MMSE, Mini-Mental State Examination; HAMA, Hamilton Anxiety Scale; HAMD-24, Hamilton Depression Scale-24; FAB, Frontal Assessment Battery. ^a^Chi-square test; ^b^One-way ANOVA; ^c^Kruskal–Wallis test; ^d^Mann–Whitney U-test; ^e^Two-sample t-test. ^*^p < 0.05, ^**^p < 0.01, ^***^p < 0.001.

### 3.2 Group differences in WM topological parameters

**Figure 1:**
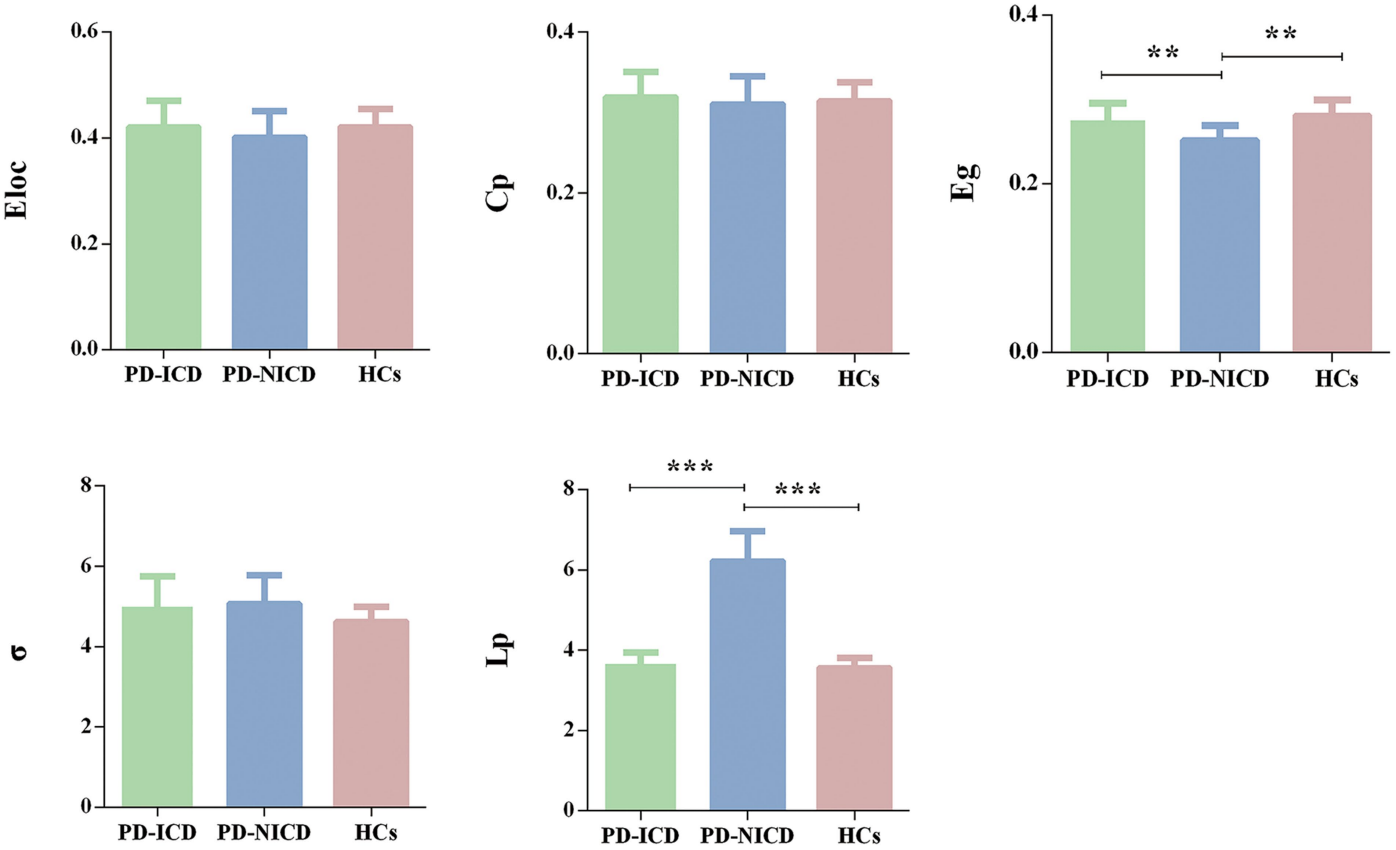
Group comparison of the global network parameters. PD-ICD group and HCs display increased Eg and reduced Lp in comparison to the PD-NICD group. PD, Parkinson’s disease; ICDs, impulse control disorders; HCs, healthy controls; Eloc, local efficiency; Cp, clustering coefficient; Eg, global efficiency; σ, small-world index; Lp, shortest path length. ^*^p < 0.05, ^**^p < 0.01, ^***^p < 0.001. *p*-values were corrected using the Bonferroni method.

For global network measures, the Eg (all *p* = 0.002) of PD-ICD patients and HCs was statistically increased, and Lp (all *p* < 0.001) was decreased when compared to the PD-NICD group (Figure 1; Table 2). PD-ICD patients, PD-NICD patients, and HCs all presented small-word organization (σ > 1) of WM structural networks. However, no significant difference was detected in Eloc, Cp, and σ among the three groups.

**Table 2:** Group differences of topological parameters among the three groups.

Topological parameters	PD-ICD	PD-NICD	HCs	*p*-value	*Post hoc* (Bonferroni correction)
***Global graph parameters***					
Eloc	0.421 ± 0.049	0.402 ± 0.049	0.422 ± 0.033	0.564	
Cp	0.320 ± 0.031	0.311 ± 0.034	0.315 ± 0.023	0.903	
Eg	0.275 ± 0.021	0.252 ± 0.017	0.281 ± 0.018	<0.001^***^	PD-ICD > PD-NICD (*p* = 0.002^**^) PD-NICD < HCs (*p* = 0.002^**^)
σ	5.000 ± 0.753	5.076 ± 0.708	4.632 ± 0.365	0.122	
Lp	3.652 ± 0.286	6.225 ± 0.740	3.572 ± 0.235	<0.001^***^	PD-ICD < PD-NICD (*p* < 0.001^***^) PD-NICD > HCs (*p* < 0.001^***^)
***Nodal graph parameters***					
***Ne***					
Middle cingulate gyrus, L	0.344 ± 0.017	0.311 ± 0.021	0.340 ± 0.022	0.002^**^	PD-ICD > PD-NICD (*p* = 0.001^**^)
Superior cerebellum, L	0.304 ± 0.047	0.243 ± 0.089	0.282 ± 0.036	0.008^**^	PD-ICD > PD-NICD (*p* = 0.011^*^)
Middle frontal gyrus, L	0.296 ± 0.019	0.281 ± 0.016	0.307 ± 0.018	<0.001^***^	PD-NICD < HCs (*p* = 0.002^**^)
Inferior parietal lobule, L	0.320 ± 0.026	0.296 ± 0.017	0.335 ± 0.018	<0.001^***^	PD-NICD < HCs (*p* < 0.001^***^)
***NLp***					
Middle cingulate gyrus, L	2.911 ± 0.145	3.226 ± 0.207	2.954 ± 0.199	<0.001^***^	PD-ICD < PD-NICD (*p* < 0.001^***^)
Fusiform gyrus, R	3.570 ± 0.278	3.346 ± 0.303	3.258 ± 0.213	0.001^**^	PD-ICD > HCs (*p* = 0.002^**^)
Inferior parietal lobule, L	3.154 ± 0.256	3.416 ± 0.169	2.995 ± 0.157	<0.001^***^	PD-NICD > HCs (*p* < 0.001^***^)

Data are presented as mean ± standard deviation. One-way ANCOVAs were used to detect the group differences in network parameters, adjusted for age, sex, education level, HAMA scores, HAMD-24 scores, and MMSE scores. *Post-hoc* analyses (corrected using Bonferroni) were then used for between-group differences. PD, Parkinson’s disease; ICDs, impulse control disorders; HCs, healthy controls; Eloc, local efficiency; Cp, clustering coefficient; Eg, global efficiency; σ, small-world index; Lp, shortest path length; Ne, nodal efficiency; NLp, nodal shortest path length; L, left; R, right. *p < 0.05, **p < 0.01, ***p < 0.001.

**Figure 2:**
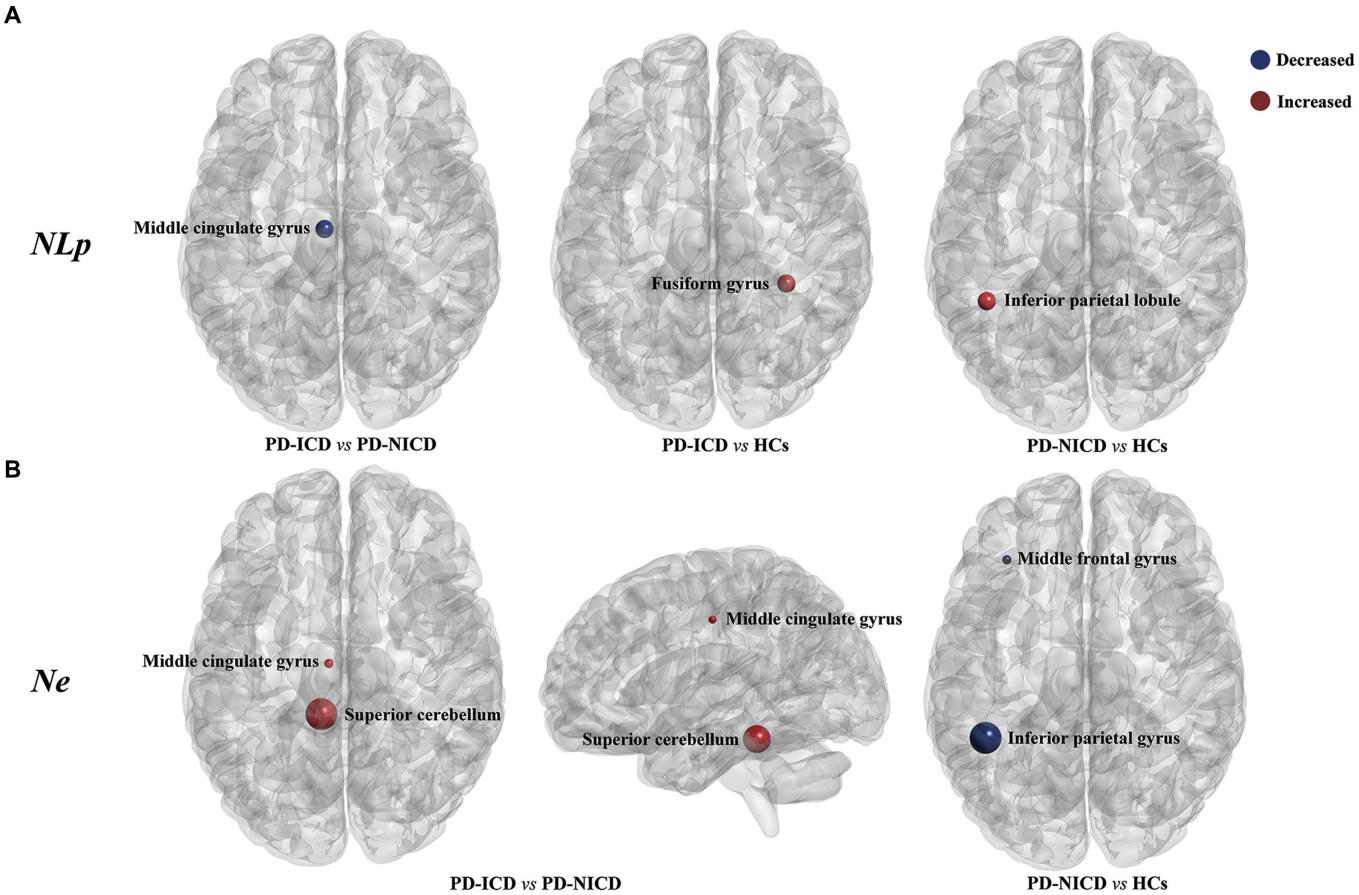
Brain regions with between-group differences in nodal parameters. **(A)** PD-ICD group shows decreased NLp (blue) in the left middle cingulate gyrus compared to the PD-NICD group. Increased NLp in ICD and NICD patients is shown in red relative to HCs, respectively, in the right fusiform gyrus and left inferior parietal lobule. **(B)** PD-ICD group shows increased Ne (red) in the left middle cingulate gyrus and left superior cerebellum compared to the PD-NICD group. Compared to HCs, PD-NICD patients displayed decreased Ne (blue) in the left middle frontal gyrus and left inferior parietal lobule. PD, Parkinson’s disease; ICDs, impulse control disorders; HCs, healthy controls; NLp, nodal shortest path length; Ne, nodal efficiency. *p*-values were corrected using the Bonferroni method. The size of the far point indicates the relative T-values from a two-sample *t-*test.

Compared to the NICD group, ICD patients manifested decreased NLp in the left MCG (*p* < 0.001) and enhanced Ne in the left MCG (*p* = 0.001) and left superior cerebellum (cerebellum 3, *p* = 0.011) at a local level. In contrast to the HCs, the ICD groups showed increased NLp in the right fusiform gyrus (*p* = 0.002). The PD-NICD patients demonstrated increased NLp in the left inferior parietal lobule (IPL, *p* < 0.001) in parallel with decreased Ne in the left IPL (*p* < 0.001) and left middle frontal gyrus (MFG, *p* = 0.002) in relation to the HCs (Figure 2; Table 2).

### 3.3 Voxel-wise GCA analysis

**Figure 3:**
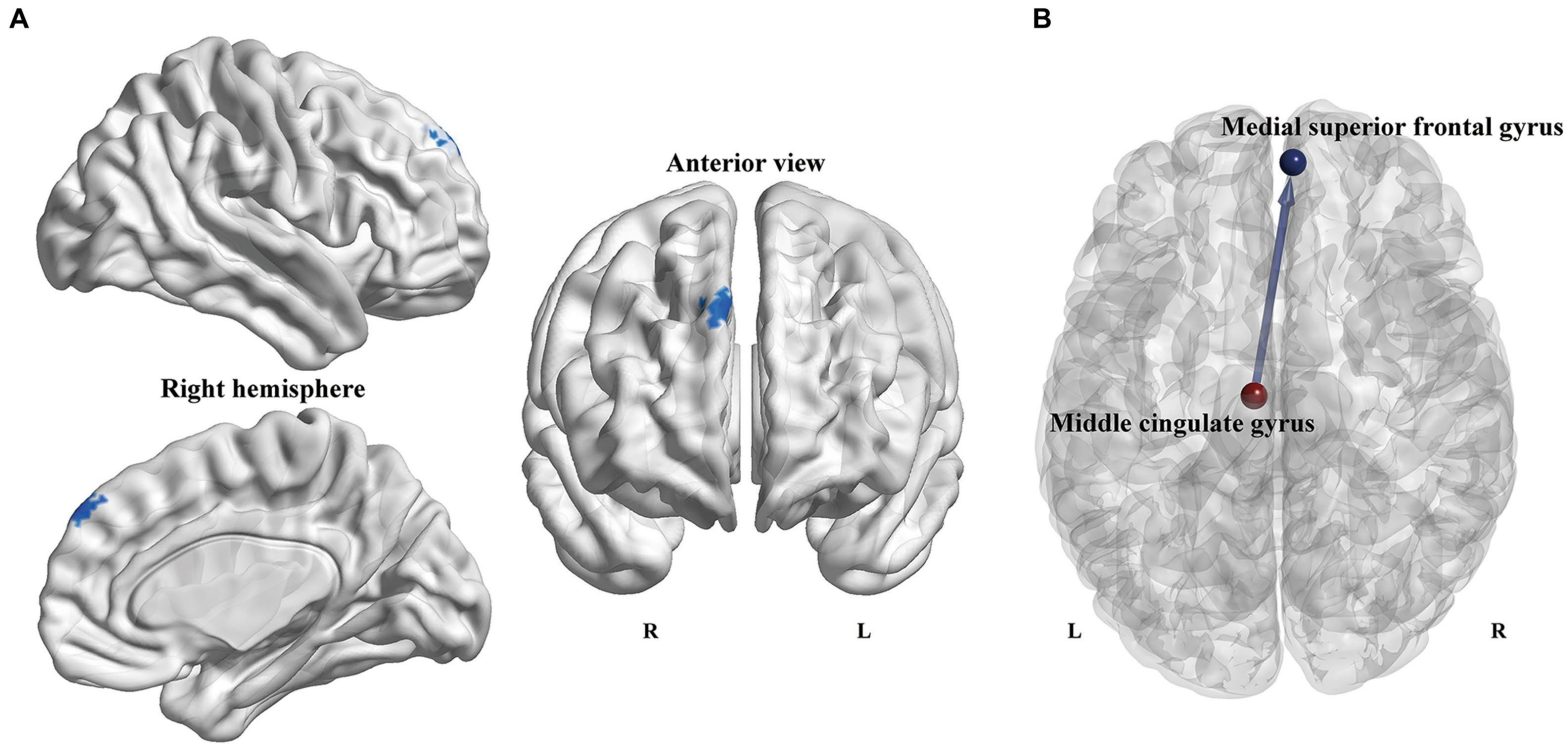
Voxel-wise GCA analysis between PD subgroups. **(A)** Statistical maps show altered causal connectivity from the left MCG to the right mSFG in the PD-ICD patients compared to the PD-NICD group. The threshold was set to *p* < 0.01 (AlphaSim correction). **(B)** Abridged general view of altered causal connectivity from the MCG between the two PD subgroups. The red node indicates the key node (left MCG) based on nodal parameters analyses. The blue node and line indicated the output brain region (right mSFG) and causal connectivity output from the left MCG, respectively. GCA, Granger causality analysis; PD, Parkinson’s disease; ICDs, impulse control disorders; MCG, middle cingulate gyrus; mSFG, medial superior frontal gyrus; L, left; R, right.

On account of the abovementioned local topological results, the left MCG and left cerebellum 3 were defined as seed regions to explore the Granger causal influence between these regions and each voxel of the whole brain. The PD-ICD patients exhibited significantly declined directional connectivity from the left MCG to the right medial superior frontal gyrus (mSFG) (MNI coordinates: x = 6, y = 54, z = 42; Peak T-value = −3.7745; voxels size: 46) in comparison to the PD-NICD patients (*p* < 0.01, AlphaSim corrected; Figure 3). No significant casual patterns were yielded with the seed as left cerebellum 3.

### 3.4 Correlations

**Figure 4:**
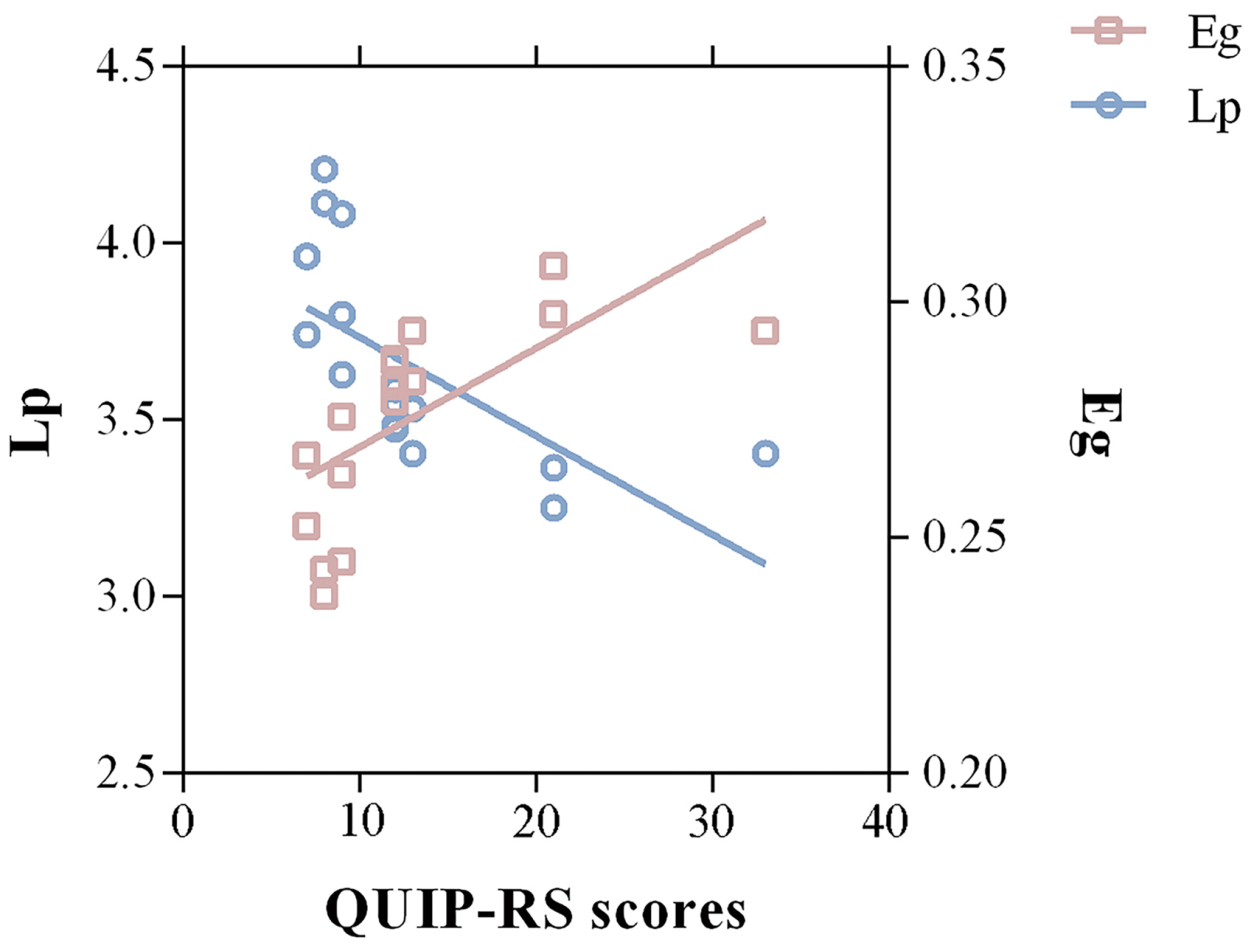
Correlations between significant graph properties and ICD symptom severity in the PD group. A negative correlation between Lp of the whole-brain structural network and QUIP-RS scores was detected in PD-ICD patients (*r* = −0.651, *p* = 0.03, FDR corrected). Meanwhile, the Eg was positively correlated with QUIP-RS scores (*r* = 0.674, *p* = 0.046, FDR corrected). Lp, shortest path length; Eg, global efficiency; QUIP-RS, Questionnaire for Impulsive-Compulsive Disorders in Parkinson’s Disease-Rating Scale.

A partial correlation analysis revealed that QUIP-RS scores were significantly correlated with Lp (*r* = −0.651, *p* = 0.03, FDR corrected) and Eg (*r* = 0.674, *p* = 0.046, FDR corrected; Figure 4). No significant correlations were detected between ICD severity and disrupted directional connectivity or nodal properties.

## 4 Discussion

In the present study, the graph theory method and GCA analysis were used to investigate the WM structural network alternation and hierarchical activity characteristics of PD-ICD patients. We observed that the Eg and Lp in ICD patients were significantly altered compared to the NICD group. The ICD patients congruously exhibited decreased NLp in the MCG, in parallel with increased Ne in the MCG and superior cerebellum. Importantly, the PD-ICD patients showed decreased directional connectivity from the left MCG to the right mSFG relative to the PD-NICD group. These findings provided novel insights into neural underpinnings of the cortical–limbic system in the pathological mechanism of PD-ICD.

The Eg, calculated as the average inverse Lp, could estimate the capability of transferring parallel information of the network. As evidenced by the increased Eg and decreased Lp, the communication of the brain network related to impulsive behaviors was enhanced, and the overall integration of the structural network was excessively optimized. This hypothesis was supported by abundant neuroimaging analyses, reporting that PD-ICD was a multidimensional disorder characterized by an abnormal overdrive of dopaminergic neuronal circuitries involving important limbic striato-pallido-thalamo-cortical loop regions (Roussakis et al., 2019). It was also reminiscent of a recent neuroimaging study emphasizing that PD-ICD patients might manifest more preserved limbic-paralimbic connection relative to other PD patients, and the more ICD severity raised, the stronger internetwork connectivity (Tessitore et al., 2017). Wise RA and Meyer GM et al. reported that although dopaminergic treatment could partially restore the normal function of the striatum, the dopaminergic agents may overdose the striatal-cortical circuit and heighten sensitization to reward cues and abnormal reward seeking, thus potentially leading to ICDs (Wise, 2009; Meyer et al., 2019). The overoptimized neural network may result in a higher susceptibility of the meso-cortico-striatal dopamine system to develop into a hyperdopaminergic state once exposed to dopaminergic treatment, which might explain the occurrence of ICD. Moreover, our correlation analysis revealed that QUIP-RS scores, which were widely applied to evaluate impulsivity severity, were significantly correlated with Eg and Lp. This further proved the previous speculation that the more optimized the structural network, the greater the impulsivity.

The NLp and Ne represented the ability of information communication between nodes. The increased Ne and decreased NLp of MCG were specific to ICD patients, demonstrating this region’s enhanced information processing and communication and highlighting its critical involvement in the onset of ICD. The MCG, commonly known as the dorsal anterior cingulate cortex, is responsible for the cognitive domain of reward processing. It is believed to be a crucial part of feedback-mediated decision-making and the modification of reward behaviors to accommodate current and predicted contexts (Vogt, 2016; Wang X. et al., 2021). The abnormal function and microstructure of the MCG are closely coupled with impulsivity (Wang Y. et al., 2021). Our study identified that the greater work efficiency in the MCG of the PD-ICD group may prompt greater monitoring of response, increased motivational salience processing, and the overevaluation of the forthcoming reward. Our hypothesis was further supported by imaging studies that found dopamine agonists could specifically drive the enhancement of the abnormal coupling of the reward circuit in PD patients who experienced ICD. This could result in dysmodulation in reward-related decision-making and a strong personality that is driven by novelty seeking (Carriere et al., 2015; Navalpotro-Gomez et al., 2020).

The current results indicated that the directional information transmission from the left MCG to the right mSFG was disrupted in PD-ICD patients. This hierarchical sequencing activity pattern suggested that the collaborative activity pattern of these two nodes was directional, and the neural activity in SFG regions was more dependent on reward-related regions. Motor response inhibition may be influenced by the attenuation of information integration from the MCG to the SFG since the frontal region is involved in the inhibitory-attentional network and manages interference inhibition and action withholding (Filip et al., 2018). The capacity to restrain improper behavior is a major function of the inhibitory system, and its impairment is considered a pivotal determinant of uncontrolled behavior. Available evidence shows that PD-ICD patients exhibit difficulty in the acquisition of stopping abilities (Leplow et al., 2017). Our findings resonated well with a previous discovery showing that PD-ICD patients had lower cortical thickness in SFG, reflecting the lack of inhibition that compulsivity is related to Roussakis et al. (2019). Furthermore, the dysfunction of the motor inhibitory mechanism could not only generate action impulsivity but also choice impulsivity. As revealed by Antonelli F et al., reduced activation of the prefrontal cortex might cause increased impulsive choices by provoking subjective devaluation of the delayed reward (Antonelli et al., 2014).

The cerebellum, conventionally regarded as a pure motor center, is currently being studied for its reward processing and cognitive functions (Schutter, 2016). Recently, studies found that the cerebellum is active during reward anticipation and has an indispensable role in encoding the expectation of reward, thus having a profound influence on impulsive behaviors (Wagner et al., 2017; Wilson et al., 2018; Geugies et al., 2022). Cerebellar processing contributes to consciously recognizing negative emotions caused by a sense of self-responsibility for an incorrect decision (Rabellino et al., 2016). Patients with abnormal cerebellar function may be unable to experience feelings of regret over their disadvantageous choices, thereby exacerbating their addictive behavior. The current research emphasized the participation of the cerebellum in ICD, showing that greater activation of the cerebellum was positively correlated with impulsivity severity (Piccoli et al., 2020; Ruitenberg et al., 2022). These paralleled results from our study, as the Ne of the superior cerebellum was elevated in the ICD group.

Thus, from our observation, the impulsivity and failure of inhibition of ICD appeared to incorporate two aspects. The first aspect was associated with the aberrant ability to deal with decision-making information and the failure to make a proper choice. Second, the motor inhibition system was disrupted, and the ability to inhibit prepotent responses and prevent inappropriate action was reduced. Our findings could provide compelling evidence for further comprehending the neurophysiological mechanism of ICD and might provide candidate brain targets for non-invasive neuromodulation. As the recent clinical trial reported, excitatory neuromodulation over the pre-supplementary motor area was successfully applied to improve response inhibition in PD-ICD patients (Mata-Marin et al., 2022).

Some limitations must be mentioned. First, this was a cross-sectional study with a limited sample size. Second, our study enrolled PD-ICD with mixed ICD subtypes, which might have neglected some findings specific to a single ICD category. It should be noted that most studies in the field regard PD-ICD as one clinical entity and do not differentiate between specific compulsive behaviors (Roussakis et al., 2019). Our finding may be driven by CS, given its large proportion. Of note, our study used on-medication scanning. Several arguments supported our experimental scheme. Technically, treatments reduced artifacts in patients. ICD was supposed to be a functional perturbation strongly associated with medication use (Carriere et al., 2015). Additionally, we acknowledge that it remains difficult to thoroughly understand the complex pathological mechanism of ICDs by simply associating the clinical scores with changes in brain regions or connectivity. In the future, we are committed to using event-related MRI in conjunction with sophisticated psychological models and behavioral tasks to continue exploring the neural bases of ICDs. There was some heterogeneity between our findings and the current published studies. We speculated that the differences in research protocols and heterogeneity in patients enrolled, with different symptoms, disease stages, number and type of ICD, and treatments, might contribute to the outcome’s diversity.

## 5 Conclusion

Impulsivity is multifaced, involving various forms of response reward evaluation, inhibition, motivation, and cognitive control (Dalley et al., 2011). Our research found that excessively strengthened topological organization of structural connectome and weakened directional connectivity in regions associated with reward processing may be associated with the occurrence of PD-ICD. These findings revealed that the neural mechanism of ICD might result from the greater activation of the reward circuit and the perturbation of the motor inhibitory system.

## Data availability statement

The raw data supporting the conclusions of this article will be made available by the authors, without undue reservation.

## Ethics statement

The studies involving humans were approved by this research was ratified by the ethics committee of the First Affiliated Hospital of Nanjing Medical University. The studies were conducted in accordance with the local legislation and institutional requirements. The participants provided their written informed consent to participate in this study.

## Author contributions

CG: Conceptualization, Formal analysis, Funding acquisition, Investigation, Methodology, Resources, Writing – original draft. HZ: Conceptualization, Investigation, Writing – review & editing. HS: Investigation, Methodology, Writing – review & editing. XC: Investigation, Writing – original draft. LW: Investigation, Methodology, Software, Writing – original draft. KZ: Conceptualization, Supervision, Visualization, Writing – review & editing. YY: Funding acquisition, Supervision, Writing – review & editing.

## Footnotes

^1^ https://www.nitrc.org/projects/gretna

^2^ http://www.nitrc.org/projects/bnv/

^3^ http://www.nitrc.org/projects/rshrf

^4^ https://www.fil.ion.ucl.ac.uk/spm/software/

^5^ http://afni.nimh.nih.gov/pub/dist/doc/manual/AlphaSim.pdf
