# Supplementary material for: Aberrant brain topological organization and granger causality connectivity in Parkinson’s disease with impulse control disorders
Source: Front Aging Neurosci. 2024 Apr 25;16:1364402. doi: 10.3389/fnagi.2024.1364402 (PMC11079187; doi:10.3389/fnagi.2024.1364402)
Supplement: Supplementary file 1 [file Data_Sheet_1.docx]

Supplementary Material

Aberrant brain topological organization and granger causality connectivity in Parkinson’s disease with impulse control disorders

# 1. Supplementary Data

## 1.1 Imaging parameters

T1-weighted anatomical images covering the whole brain were obtained by the volumetric 3D magnetization-prepared rapid gradient-echo (MP-RAGE) sequence: repetition time [TR] = 1900 ms, echo time [TE] = 2.95 ms, flip angle [FA] = 9°, thickness = 1 mm, slices = 160, field of view [FOV] = 230 × 230 mm^2^, acquisition matrix = 256 × 256 and voxel size = 1 × 1 × 1 mm^3^. The DTI data was acquired by spin echo planner imaging sequence, with the following acquisition parameters: TR = 9800 ms, TE = 95 ms, FOV = 256 × 256 mm^2^, number of excitations (NEX) = 1, matrix = 128 × 128, slice thickness = 2mm and slice gap = 0 mm. Diffusion gradients were applied in 30 non-collinear directions with a *b* factor of 1000s/mm^2^ after an acquisition without diffusion weighting (*b* = 0 s/mm^2^) for reference. Resting-state functional images were acquired using an echo-planner imaging (EPI) sequence with the following scan parameters: TR = 2000 ms, TE = 21 ms, FA = 90°, FOV = 256 × 256 mm^2^, in-plane matrix = 64 × 64, slice = 35, slice thickness = 3mm, no slice gap, total volumes = 240.

**1.2 MRI data preprocessing**

The DTI data were preprocessed with PANDA toolbox (<http://www.nitrc.org/projects/panda>) based on FMRIB Software Library (FSL 5.0; <http://www.nitrc.org/projects/fsl>). The procedures included brain extraction, realignment, eddy current, motion artifact correction, fractional anisotropy (FA) calculation, and diffusion tensor tractography. To construct whole brain fiber tractography, we employed deterministic tractographic method that used the fiber assignment by continuous tracking (FACT) algorithm (Nigro et al., 2016). The reconstruction of each fiber was interrupted if it turning angle was greater than 45° or reach voxel with a FA value less than 0.2.

The rs-fMRI data preprocessing was performed using Data Processing Assistant for rs-fMRI (DPARSF). The primary steps were as follows: ① the removal of the first 10 time points; ② slice timing correction; ③ realign; ④ spatial normalization by DARTEL; ⑤ spatially smoothing with a Gaussian kernel of 6 × 6 × 6 mm^3^ full-width at half -maximum; ⑥ nuisance signal regression; ⑦ temporal filtering (0.01-0.08 Hz) and linearly detrending.

**Reference:**

Nigro, S., Riccelli, R., Passamonti, L., Arabia, G., Morelli, M., Nistico, R., et al. (2016). Characterizing structural neural networks in de novo Parkinson disease patients using diffusion tensor imaging. *Hum Brain Mapp* 37(12)**,** 4500-4510. doi: 10.1002/hbm.23324.
